# Direct Versus Video Laryngoscopy in Emergency Intubation: A Randomized Control Trial Study

**DOI:** 10.30476/BEAT.2021.89922.1240

**Published:** 2021-07

**Authors:** Pitsucha Sanguanwit, Chaiyaporn Yuksen, Nishapa Laowattana

**Affiliations:** 1 *Department of Emergency Medicine, Faculty of Medicine, Ramathibodi Hospital, Mahidol University, Bangkok, Thailand*

**Keywords:** Video laryngoscopy, Macintosh direct laryngoscope, Emergency department

## Abstract

**Objective:**

To compare the intubation success rate of the first attempt between Video Laryngoscopy (VDL) and Direct Laryngoscopy (DL) in the emergency department (ED).

**Methods:**

This is a study of a randomized control trial includes the patients with acute respiratory failure and the emergency physician who intended to perform intubation in the ED from July 2015 to June 2016. We were selected the patients randomly by the sequentially numbered opaque sealed envelopes technique and were assigned to undergo the first attempt of either VDL (n=78) or DL (n=80). We collected the data information regarding the demographic characteristics, predictors of difficult intubation, rapid sequence intubation, attempt, Cormack–Lehane view, and immediate complications.

**Results:**

The success of VDL in the first attempt was 73.1%, which were tended to be better than DL (58.8%) (*p*=0.060). Glottis view (Cormack–Lehane view 1–2) of VDL was significantly better (88.5%) than of DL (72.5%) (*p*=0.010). The immediate complications were not different.

**Conclusions:**

VDL showed a trend of better success than DL. VDL can increase the first-attempt intubation success and provide a better glottis view in emergency intubation.

**Trial registration:**

The trial was registered in the Thai Clinical Trial Registry, identifier TCTR 20200503003. Registered 16 June 2020, ‘Retrospectively registered’, http://www.clinicaltrials.in.th/index.php?tp=regtrials&menu=trialsearch&smenu=fulltext&task=search&task2=view1&id=6186

## Introduction

Airway management is an important procedure that must be prioritized in the ED. Advanced airway management and tracheal intubation are procedures required to prevent airway compromise and respiratory failure. Rapid and effective airway management can prevent patient deterioration and cardiac arrest.

Direct laryngoscopy (DL) is a standard method used in general tracheal intubation [1]. However, this method may not result in successful intubation and difficult-to-intubation situations, because it does not provide alignment to visualize the vocal cord due to the failure rate of the first successful intubation. Thus, it is necessary to assess such difficult conditions before performing intubation because increased intubation attempts might increase the risk of hypoxemia cause further complications of tracheal intubation [2, 3]. The intubation difficulties evaluation would help the physician understand the risk and thus plan and set up airway management for the patient.

Current laryngoscopes have been advanced to visually screen the epiglottis and the vocal cord during the endotracheal tube insertion which is a technique known as ‘video laryngoscopy,’ (VDL) and is another choice for patients with difficult intubation [4]. Recent studies compared the efficiency between DL and VDL and have reported the controversial results of a better method which could not be confirmed for general patients [5-9].

VDL is another choice of intubation assistance in the ED that requires experience and training. However, the success in the first attempt of intubation between VDL and DL was not different [10], and VDL required more time to achieve success in the first attempt than the DL method [10-12].

Other studies have reported that VDL may improve intubation success in the first attempt and decrease complications during the ED’s intubation period especially in patients with difficult airways [10-12]. However, some data showed that VDL require more time to achieve success, and the success was also not different [13, 14]. Therefore, this study’s objective is to compare DL and VDL’s intubation success in the ED.

## Materials and Methods

*Study Design*

This study was a randomized controlled trial in respiratory failure patients who visited to the ED of Ramathibodi hospital from July 2015 to June 2016. We used the sequentially numbered opaque sealed envelopes (SNOSE) technique to random the patients and were assigned to undergo the first intubation with VDL (GlideScope) or DL. We also collected the baseline prediction factor of difficult intubation, gender, age, predictors of difficult intubation, time to successful intubation, and Cormack–Lehane view.

The ethics committee approved this study of the Faculty of Medicine, Ramathibodi Hospital, Mahidol University, in January 2015. The trial was registered in the Thai Clinical Trial Registry, identifier TCTR 20200503003

*Participants*

First, second, and third-year emergency residents or emergency attending staff were defined as intubation experiences. Sixth-year medical students (under the supervision of emergency residents and emergency attending staff) were defined as not having expertise in intubation. All participants received training in laryngoscopy by using manikin before signing an agreement to participate in the research project.

*Study Setting and Population*

We were included the acute respiratory failure patients and the emergency physician who intended to perform intubation in the ED of the University Hospital, Faculty of Medicine, Ramathibodi Hospital, Mahidol University as a tertiary care university hospital.

*Inclusion and Exclusion Criteria*

The inclusion criteria were patients more than 18 years olf and who met the intubation criteria. The exclusion criteria were patients who had no signs of undergoing resuscitation and patients who were at the end of life care.

*Randomization Allocation and Concealment*

We randomized the patients by using the sealed opaque envelope in a block of 10 with SNOSE before enrolment in the ED.

*Data Collection and Measurement*

Participants were randomized to use for intubation either the VDL or the DL. The research protocol was an open-label study for intubation assigned from the randomization. Both groups of enrolled patients were also receiving other standard treatments according to Ramathibodi Hospital emergency airway protocol. The researchers were not involved in other treatment procedures.

Baseline characteristics such as age, gender, predictors of the difficult airway (appearance, evaluation, Mallampati score, obstruction, and neck mobility), rapid sequence intubation (RSI), utilization time for intubation attempts, and immediate complications were collected at the time of enrolment.

*Clinical Outcome*

We were recorded the first attempt of intubation success to the primary outcome. The first success of intubation attempt was defined as the utilized time from the blade’s open-mount application until passing the endotracheal tube to the vocal cord with a blow cuff pressure, and in oesophageal intubate, we defined the attempt as a failure to intubation. The secondary outcomes were the second attempt intubation success rate and the Cormack–Lehane view during the intubation attempt.

*Sample Size and Statistical Analysis*

The sample size was calculated by using the equation for two independent proportions in comparison to the previous data of Hyuk Joong Choi *et al*., [5]. The first pass success rate of intubation was 50.4% by the direct laryngoscope, whereas the video laryngoscope was 80%. We were applied the data to the research equation of cohort for binary data. We present the p1 (Exposure) = 0.54, p2 (Un-exposure)=0.8, ratio (r)=1. Using a 2-side type 1 error=0.05 power 80%. We required a sample size of 39 participants in each group.

**Fig. 1:**
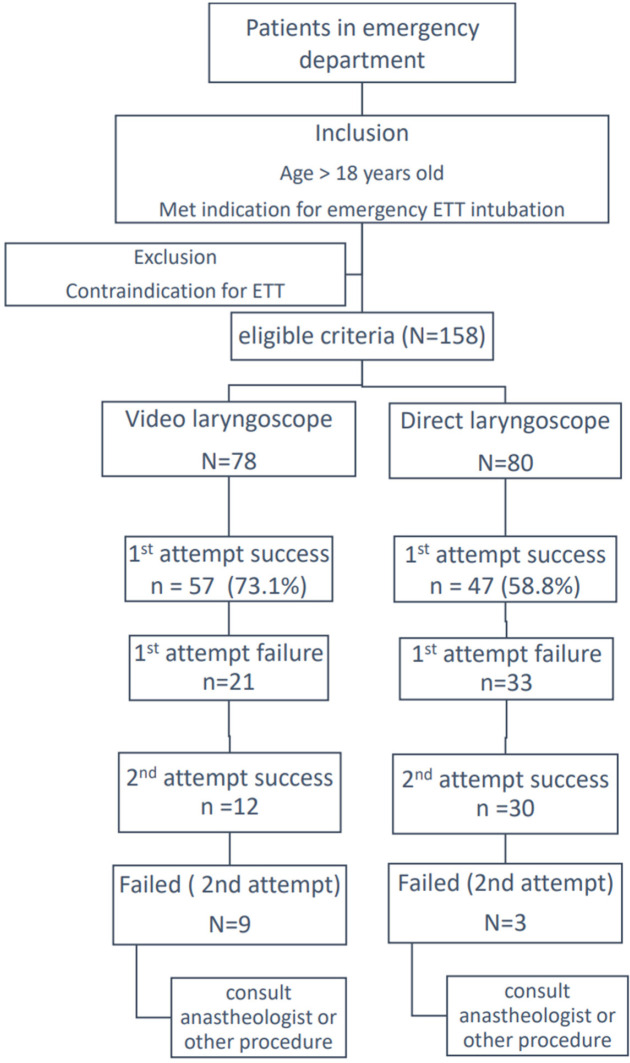
Patient flow chart

Therefore, we conducted an intention-to-treat analysis. Descriptive analysis was used for general characteristic data. Continuous variables were represented by mean±SD (in the normal distribution or median and IQR) by using a non-parametric test, independent t-test, or Mann–Whitney U test as appropriate. Categorical data were represented by percentage using the Chi-squared test or Fisher’s exact test as appropriate. We performed all data analyses by using Stata version 12 and SPSS version 18 (Figure 1).

## Results

A prospective, randomized crossover study using manikin conducted at Ramathibodi Hospital in 2013 demonstrated that the laryngeal view (Cormack–Lehane classification) from the video laryngoscope was better than that from the direct laryngoscope (100% vs. 85%, *p*<0.010), especially in the group with less experience in intubation (medical students) (100% vs. 80.39%; *p*<0.010).

A total of 158 patients were included by randomization between July 2015 and June 2016 in this study. Of these patients, 78 underwent intubation by VDL, and 80 received intubation by DL (Figure 1).

Table 1 presented the baseline prediction factor of difficult intubation. There were 84 men patients (53.2%). Gender and predictors of difficult intubation includes appearance, evaluation, Mallampati score, obstruction, and neck mobilization were generally similar between both groups, except age with 73±12.9 years in the video laryngoscope group vs. 65±17.2 years in the DL group (*p*=0.01). RSI technique was similar between both groups (62.8% in the VDL group vs. 60% in the DL group). The median time of the first intubation success was not different. 

**Table 1:** Baseline prediction factor of difficult intubation

**Prediction factor of difficult intubation**	**VDL** ^d^ ** (n=78)**	**DL** ^e^ ** (n=80)**	***p*** ** value**
Gender: Male; n (%)^a^	44 (57%)	40 (50%)	0.41
Age_yr: mean±SD^b^	73±12.9	65±17.2	0.01
Difficult intubation (abnormal LEMON)			
External appearance (abnormal)	11 (14.1%)	11 (13.8%)	0.95
Evaluate 3-3-2 abnormal)	9 (11.5%)	10 (12.5%)	0.94
Mallampati score			
Grade 1	5 (6.4%)	1 (1.3%)	0.49
Grade 2	6 (7.7%)	7 (8.8%)	
Grade 3	4 (5.1%)	6 (7.5%)	
Grade 4	4 (5.1%)	6 (7.5%)	
N/A	59 (75.6%)	60 (75%)	
Obstruction (abnormal)	2 (2.6%)	0	0.12
Neck mobility (abnormal)	5 (6.4%)	4 (5%)	0.74
Rapid sequence intubation	49 (62.8%)	48 (60%)	0.72
Median time of successful intubation(s); Med [Q1, Q3]^c^	15 [10, 30] N=36	5 [10, 30] N=28	0.53
Cormack–Lehane view			
View 1–2	127 (84.0%)	69 (88.5%)	58 (72.5%)
View 3–4	31 (16.0%)	9 (11.5%)	22 (27.5%)

Table 2 showed the success of intubation in the first attempt success. Overall, 104 (65.8%) patients achieved success in the first attempt of intubation. VDL showed a higher success rate than direct laryngoscope [57 (73.1%) vs 47 (58.8%)] patients (*p*=0.06). In the subgroups of participants, VDL showed a significantly higher success rate than direct laryngoscopy [46/62 (74.2%) vs. 32/57 (56.1%) patients (*p*=0.01) in the non-experienced group (sixth-year medical students)]. However, this result was similar in the experienced group (resident trainee and staff) [11/16 (68.8%) vs. 15/32 (65.2%)].

**Table 2:** The success of intubation

**Success of intubation**	**Overall (n=158)**	**VDL** ^c^ ** (n=78) **	**DL** ^d^ ** (n=80)**	*p* ** value**
1^st^ attempt success N (%)	104/158 (65.8%)	57 (73.1%)	47 (58.8%)	0.06^a^
Experience				
Non-experienced (119)	78/119 (65.5%)	46/62 (74.2%)	32/57 (56.1%)	0.03^a^
Experienced (39)	26/39 (66.7%)	11/16 (68.8%)	15/23 (65.2%)	0.82
RSI technique				
RSI^e^ (97)	74/97 (76.3%)	42/49 (85.7%)	32/48 (66.7%)	0.03^a^
Non-RSI (61)	30/61 (49.2%)	15/29 (51.7%)	15/32 (46.9%)	0.71
2^nd^ attempt success N (%)	42 (77.8%)	12 (57.1%)	30 (90.9%)	<0.01^a^
Non-experience (5)	4/5 (80%)	2/3 (66.7%)	2/2 (100%)	1.00^b^
Experience (49)	38/49 (77.6%)	10/18 (55.6%)	28/31 (90.3%)	<0.01^a^

^a^Chi-square test; ^b^Fisher’s exact test, *p*<0.05 was considered statistically significant; ^c^VDL: video laryngoscope; ^d^DL: direct laryngoscope; ^e^RSI: rapid sequence intubation

Overall second attempt success of intubation was achieved in 42/54 (77.8%) patients (Table 2). VDL significantly had lower success rates than DL [12 (57.1%) vs 30 (90.9%) patients (*p*=0.01)]. In the participant’s subgroups, VDL showed significantly lower success rates than DL [10/18 (55.6%) vs. 28/31 (90.3 %) patients (*p*<0.01) in the experienced group (resident trainee and staff)], but this result was similar in the non-experienced group (sixth-year medical students) [2/3 (66.7 %) vs. 2/2 (100.0%)]. The Cormack–Lehane view was significantly better (Cormack–Lehane view 1–2) than that by DL group [69 (88.5%) vs. 58 (72.5%) patients (*p*=0.01)] during VDL intubation.

The intra-oral bleeding and broken tooth were similar between both groups in complications after intubation with 2.5% in the VDL and 3.2% in the DL. In the DL group, oesophageal intubation was found only in one case. There were no severe life-threatening complications such as cardiac arrest in both groups.

**Fig. 2:**
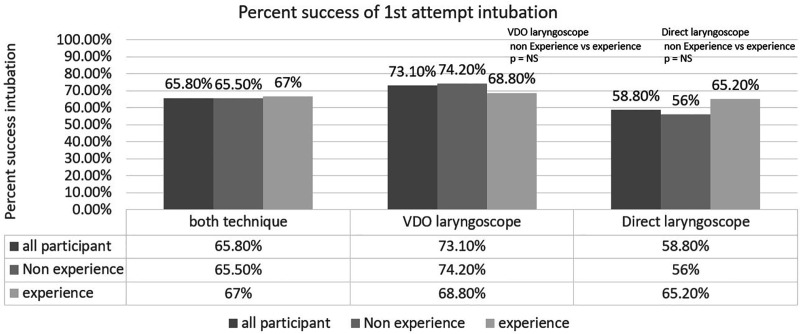
Percentage success of first attempt intubation according to experience of participants

Comparison between the non-experienced and experienced group revealed similar first attempt success intubation rates in both video laryngoscope and direct laryngoscope groups for each equipment (Figure 2).

**Fig. 3:**
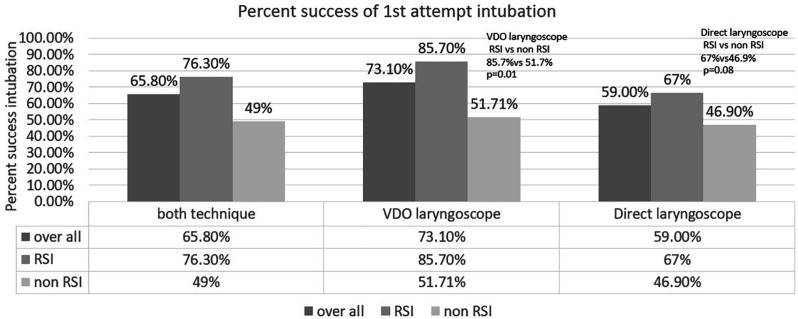
Percentage success of first attempt intubation according to use of rapid sequence intubation (RSI) technique

Also, the present study showed that drug-assisted intubation or RSI technique also improved the success of the first attempt intubation of VDL than DL. (85.7% vs. 51.7%, *p*=0.01, Figure 3).

**Fig. 4.**
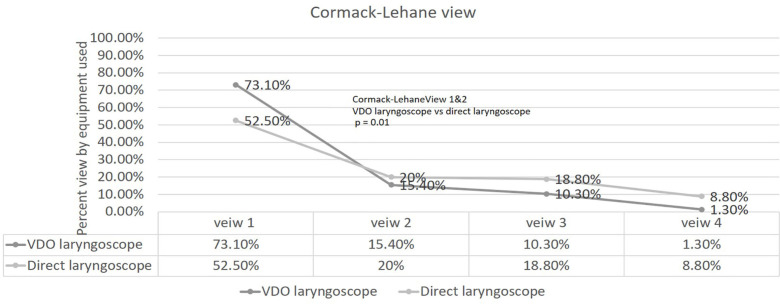
Cormack–Lehane view according to the equipment used P <0.05 was considered statistically significant

The present study data showed that VDL provided a significantly better Cormack–Lehane view than DL (Figure 4).

## Discussion

In this study, the overall success of VDL in the first attempt trend to better. Thus, VDL improves the success for difficult-to-intubate patients and non-difficult airways. The study of Liu *et al*., [15] reported a similar outcome. In the subgroups of participants, the non-experienced groups could use VDL with higher first-attempt success intubation than those by using the DL (Table 2), which is similar to the result reported by Hendrik E *et al*., [16]. After hands-on training, the VDL appears to be usable by non-experienced participants. In our study, the participants were classified according to their experience in intubation (resident trainee and staff had an experience of >3 years in DL) and no experience in intubation (the sixth-year medical student had an experience of <1 year in DL). They had less experience in VDL before receiving training for laryngoscopy by using manikin for this study. 

However, we found a different outcome in the experienced group which showed that VDL did not improve the intubation’s first attempt success rate. Jiang *et al*., [17] and Sulagna *et al*., [10] have reported that VDL did not improve intubation’s success than DL in both experienced and non-experienced groups. Recent data are controversial, which may due to confounding factors such as patient cardiopulmonary-hypoxia reserve, secretions or blood that can impair the video laryngoscope view [18, 19], RSI technique, drug selection, and operators’ experience [20].

Comparison between the non-experienced and experienced group revealed similar first attempt success intubation rates in both video laryngoscope and direct laryngoscope groups for each equipment.

6^th^-year medical student (non-experienced) had experience in intubation of less than 1 year. Experiences resident trainee and staff had to experience intubation of more than 3 years. Then, *p*<0.05 was considered statistically significant. Also, the present study showed that drug-assisted intubation or RSI technique also improved the success of the first attempt intubation of VDL than DL.

Murrell *et al*., [21] and Manoach *et al*., [22] assessed the effectiveness of VDL and reported this method could improve laryngeal views in patients with difficult airway such as those with macroglossia, obesity, and limitation of neck movement [21, 22]. 

The present study data showed that VDL provided a significantly better Cormack–Lehane view than DL. Our study also showed that the median time to successful intubation between VDL and DL were similar which is different from reports of Kim *et al*., [23] and Platts-Mills *et al*., [13]. These researchers reported successful intubation by VDL more time than DL [18]. There are also very few complications in this study such as oropharyngeal injury, which is similar between the groups. In addition, no severe or life-threatening complications were observed in both groups.

VDL will increase the first-attempt intubation success than DL in emergencies, especially in non-experience groups, and it also improved the Cormack–Lehane view (view1–2). The VL is a device that physicians can use with any degree of experience. Therefore, it is an alternative device that can improve the success of endotracheal intubation.

## Limitations

They were some limitations in our study. First, our data were derived from a single center. Second, open-label interventions may have a bias for other treatments and outcomes. Third, emergencies that may have affected the exact time to intubation and time-record were limited only in 64/104 (61.5%) first attempt in intubation. Fourth, the view of direct laryngoscope by Cormack–Lehane was recorded by one operator, whereas the video laryngoscope was recorded and reviewed by two operators. Fifth, some confounding variables were not collected in this study, such as patient’s cardiopulmonary-hypoxia reserve, secretions or blood that can impair video laryngoscope view, sedation, and paralysis drug selection, and we cannot explain why successful of second attempts by video laryngoscope was decreasing. Finally, the sixth-year medical students and early-year resident trainees of our institute did not have much experience in video laryngoscopy, which may have affected the intubation of the first attempt success. Nevertheless, our study showed that the resulted of VDL has a better success rates than DL even in cases of non-difficult airway predictors and non-experienced participants.

## Consent for publication:

Not applicable.

## Disclosure:

The abstract was presented in “38th International Symposium on IntensiveCare and Emergency Medicine.” 2018.

## Availability of data and materials:

The datasets analyzed during the current study are not publicly available due to privacy issues but are available from the corresponding author upon reasonable request.

## Funding support:

We did not receive any support from sponsor. No institutions or company involved in any aspect of the design and conduct of the study or protocol.

## List of Abbreviations

VDL: video laryngoscope, DL: direct laryngoscope, SNOSE: Sequentially numbered opaque sealed envelopes, RSI: rapid sequence intubation, P <0.05 was considered statistically significant
